# Closing the Harm Reduction Gap: EMPOWERing People Who Smoke

**DOI:** 10.7759/cureus.111096

**Published:** 2026-06-18

**Authors:** Lindsay Reese, Marina A Murphy, Kim Murray

**Affiliations:** 1 Scientific Affairs, HAYPP Group, Stockholm, SWE; 2 Scientific Affairs, HAYPP Group, Milton Keynes, GBR; 3 Tobacco Harm Reduction, MN Smoke Free Alliance, Brainerd, USA

**Keywords:** health equity, health policy, nicotine, patient-centered care, public policy, risk reduction behavior, smoking

## Abstract

Global tobacco control efforts, influenced substantially by the World Health Organization Framework Convention on Tobacco Control and the MPOWER package, have lowered smoking prevalence in some populations but have failed to address the lived realities of the people who continue to smoke. These individuals increasingly come from communities marked by socioeconomic disadvantage, lower educational attainment, and limited access to healthcare, contexts where structural constraints, chronic stress, and reduced opportunity shape tobacco use far more powerfully than individual “choice.” Yet tobacco control frameworks continue to prioritize abstinence-only strategies, punitive measures, and restrictive regulatory environments that rarely reflect the perspectives or needs of the people most affected. Countries are praised for adopting increasingly restrictive policies, with little attention given to the actual reductions in smoking rates.

This editorial argues that further decreases in smoking rates can be achieved if the focus shifts from moralizing nicotine use in any form to empowering people who smoke with practical, science-based harm reduction tools. Noncombustible nicotine products that reduce toxicant exposure are often dismissed or prohibited despite growing evidence of their value for people unlikely to achieve abstinence. This disconnect widens health inequities and alienates those currently navigating stigma, misinformation, and limited cessation support.

We propose a real-world EMPOWER framework that integrates evidence, pragmatism, risk-proportionate regulation, and person-centered communication. To reduce smoking-related harm more effectively, policy must move beyond ideology and prioritize autonomy, equity, and practical harm reduction.

## Editorial

There have been more than 20 years of global tobacco control efforts, including the 2005 establishment of the World Health Organization Framework Convention on Tobacco Control (WHO FCTC) and the implementation of MPOWER three years later [1]. The MPOWER package consists of six evidence-based tobacco control measures designed to help countries reduce tobacco use. Each letter represents one intervention and a possible number of points, with a maximum score of 34 [2]: Monitor tobacco use and prevention policies (4 points); Protect people from tobacco smoke (5 points); Offer help to quit tobacco use (5 points); Warn about the dangers of tobacco (5 points); Enforce bans on tobacco advertising, promotion, and sponsorship (5 points); and Raise taxes on tobacco (10 points).

Two countries, Türkiye and Brazil, have achieved the highest possible MPOWER score of 34, reflecting full implementation of WHO-recommended tobacco control measures across all six domains. However, a perfect MPOWER score does not mean low smoking prevalence. The countries’ estimated 2024 smoking rates were 31.1% and 11.9%, respectively [3]. While Brazil is faring better, its Ministry of Health reported that 2024 saw a rise in the proportion of adults who smoke in major cities, from 9.3% to 11.6% [4]. MPOWER only captures the strength of policy implementation; smoking prevalence also reflects policy enforcement and population-specific factors.

The limits of MPOWER and the need for reframing

In 2000, an estimated 1.379 billion people were using one or more tobacco products, and in 2024 the figure had decreased to 1.2 billion (180 million fewer) [3]. There is no doubt that tobacco control measures have helped prevent people from taking up smoking and reduced secondhand smoke exposure, but smoking-related disease also kills an estimated 7 million people annually [5]. It is therefore possible that much of the decrease is due to death rather than cessation. These declines should not be automatically interpreted as evidence that current policies are adequately supporting those who remain at the highest risk.

The number of people who smoke remains substantial, and the decline in smoking prevalence has slowed [6]. Global forecasting indicates that existing tobacco control policies must at least be maintained for smoking prevalence to continue declining as expected, but also that substantial additional health gains would require a faster reduction in smoking prevalence than current trajectories suggest [7]. Technological innovations in the last 20 years have produced newer products that deliver nicotine without smoke (e.g., e-cigarettes, nicotine pouches, and heated tobacco products), and these products reduce user exposure to many or most of the harmful chemicals in smoke that cause disease [8-10]. Nicotine is dependence-forming and not risk-free, but it is also not the driver of smoking-related disease [11]. Unfortunately for the 1.2 billion people who smoke, these products are demonized as a gateway to smoking or as perpetuating nicotine addiction. This leads to confusion around the molecule itself. For example, a 2022 study of more than 9,000 U.S. adults who smoked found that those who considered nicotine a primary cause of smoking-related disease were less likely to use alternative nicotine products and had lower smoking cessation success [12].

Despite being one of the most harmful consumer products ever created, cigarettes remain easily accessible in nearly every country in the world. They are sold in convenience stores, gas stations, supermarkets, corner kiosks, and even pharmacies, where they generate hundreds of billions in tax revenue per year [13]. Their ubiquitous availability stands in stark contrast to the increasingly restrictive regulatory landscape surrounding lower-risk nicotine products. Many countries have banned products such as nicotine pouches, e-liquids, e-cigarettes, or heated tobacco devices [14-16], even though these products do not involve combustion and expose users to far fewer toxicants than cigarettes [17]. Neither country with perfect MPOWER scores (Brazil and Türkiye) allows the sale of e-cigarettes, heated tobacco products, or nicotine pouches. Restrictions and bans are typically justified by precautionary public health principles, including concerns about youth uptake, dependence, and limited long-term safety evidence [18]; however, evaluations of these policies suggest mixed outcomes, including reductions in vaping alongside potential substitution effects [19] and the emergence of illicit markets [20].

This regulatory asymmetry has profound public health implications. When the most lethal form of nicotine delivery remains widely sold but alternatives are prohibited or heavily restricted, people continue using the very product that causes nearly all smoking-related disease and death. Such policies undermine harm reduction strategies and contradict fundamental principles of proportionality: regulating products according to their actual level of risk. If the goal is truly to reduce smoking-related harm, it is difficult to justify a world in which cigarettes are available on virtually every street corner while alternative nicotine products are rendered inaccessible in a growing number of countries.

The WHO FCTC agenda prioritizes an abstinence-only framework and the pursuit of a nicotine-free world. The concept of a “nicotine-free world” is rooted in idealism that disregards the complex social, cultural, and neurobiological underpinnings of nicotine consumption [21]. By moralizing nicotine use and conflating it with tobacco-related harm, current policy risks undermining pragmatic approaches to reducing disease and death and alienating those it seeks to help.

It is time to ask: Are current strategies sufficient, or is a new approach required to maximize public health benefits? Tobacco control should evolve beyond punitive measures and consider complementary ways to decrease smoking [22,23].

All stick and no carrot: the case for EMPOWERment

The WHO defines empowerment as “a process through which people gain greater control over decisions and actions affecting their health” [24]. In contrast, the MPOWER package relies on restrictions, taxation, and warnings and offers little in the way of positive support. People who smoke are left navigating a landscape of higher costs, prohibitions, and stigma rather than being equipped with accurate information about the risks and benefits of different quitting aids and nicotine-delivery products.

The “stick” of MPOWER has driven smoking rates down, but the returns are diminishing. We propose that it is time for a “carrot.” A different EMPOWER framework could include seven new principles. Evidence-based: Ground interventions in science instead of ideology, for example, by regulating products based on their toxicity [25]. Messaging: Communicate clearly about relative risks to counter misinformation and moralization. The U.S. Food and Drug Administration allows manufacturers to apply for authorization to include modified-risk claims on packaging [26]. Pragmatism: Prioritize realistic, person-centered strategies that acknowledge the lived experiences of people who smoke(d) and support incremental progress rather than all-or-nothing expectations. Similar approaches have been proposed for healthcare settings [27]. Options: Rather than blanket bans, allow access to products that have been shown to help people move away from smoking. Countries that do this have seen other nicotine products displace cigarettes [23]. Well-being: Focus on improving health outcomes holistically, recognizing that adults have autonomy to use nicotine in less harmful forms [21]. Engagement: Foster collaboration among clinicians, communities, and patients to build trust and shared responsibility for harm reduction. In the absence of supportive policy frameworks, healthcare providers can take the initiative to empower their patients using curated information sites [28]. Responsibility: Enact age restrictions, adhere to product standards, implement marketing controls, and perform regular surveillance of youth uptake.

**Figure 1:**
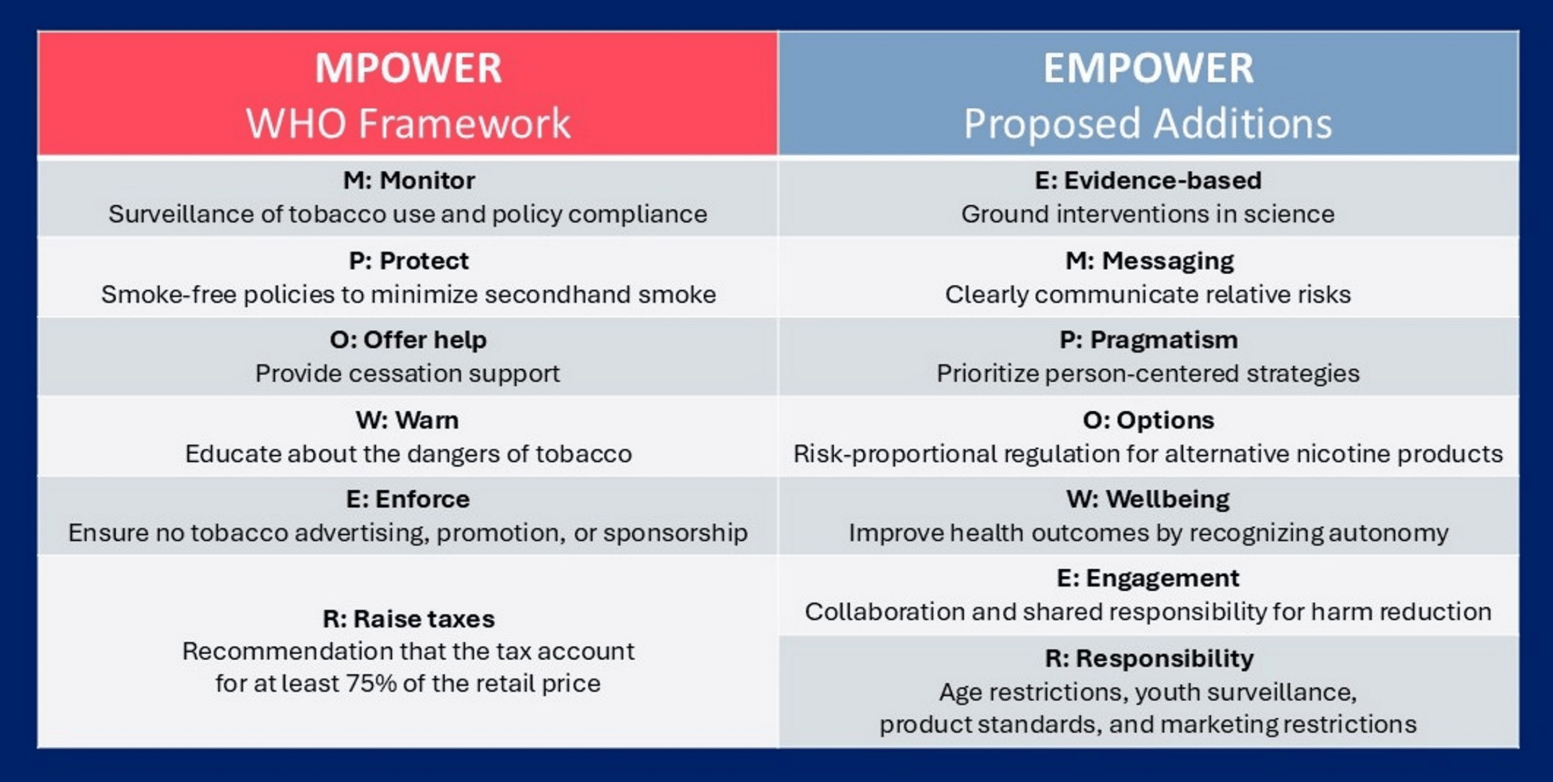
MPOWER/EMPOWER Comparison The WHO MPOWER package (left) assigns up to 34 points based on the adoption of restrictive policies. The proposed EMPOWER additions (right) complement existing controls with person-centered harm reduction principles and risk-proportionate regulation. The metrics of EMPOWER are sustained reductions in combustible tobacco use and smoking-related harm.

There would be no points assigned in the EMPOWER framework. The only metric worth measuring is the acceleration of sustained reductions in combustible tobacco use and smoking-related harm, especially among groups for whom current policies have delivered the fewest benefits. A comparison of MPOWER and EMPOWER is shown in Figure 1.

Changing demographics of smoking reveal growing inequity

A new approach is necessary because, while smoking rates have declined dramatically for the wealthiest and most educated, stark demographic differences remain. When considering the numbers, it is important to note that they do not capture populations that are systematically excluded from household-based surveillance, including people who are incarcerated, unhoused, or living in other institutional or unstable settings. This likely underestimates smoking prevalence in the most marginalized groups.

Income

While smoking has decreased globally, socioeconomic disparities have grown. The WHO claims that “lower-income smokers benefit disproportionately in terms of health gains and income retention from reduced tobacco use” [13], but the data suggest otherwise.

In the European Union, the smoking rate among low-income residents is double that of high-income residents (40% vs. 19%) [29]. In England, lower socioeconomic position is associated with a higher likelihood of smoking, greater tobacco addiction, and lower motivation to quit. The smoking prevalence rate was 10.9% for those with household incomes greater than £50,000, compared to 22.0% for those with incomes of £13,500-24,999 [30]. A similar pattern is found in the U.S. According to data collected between 2011 and 2014, approximately 36.0% of people below the poverty line smoked cigarettes, compared to 20.9% of those at or above it [31]. More recent surveillance collected since alternative products entered the U.S. market shows substantial absolute declines in smoking across all income groups but persistent relative socioeconomic disparities. Using newer indicators of socioeconomic position, 15.8% of adults classified as having low income smoked regularly, compared with 5.6% among those with high income [32]. There is a clear pattern across multiple countries: smoking has become increasingly concentrated among people with fewer economic resources, even as population-level prevalence declines.

Age

Age patterns in overall tobacco use show a clear midlife peak. The WHO estimated that in 2022, the age group with the highest prevalence of tobacco use was adults aged 45-54 (26.4%), followed by ages 55-64 (24.8%), 35-44 (24.7%), and 65-74 (21.1%) [6]. The lowest estimated rates were in the ≥85 and 15-24 age groups (12.9% and 13.3%, respectively).

A 2025 cross-national analysis of nationally representative adult samples from 22 countries found that cigarette smoking prevalence differs markedly across age groups, with higher prevalence consistently observed among middle-aged and older adults than among younger adults, although the exact gradients vary by country and context [33]. Data from the 2022 National Health Interview Survey (NHIS) showed that the highest rate of current cigarette smoking was among U.S. adults aged 45-64 (15.1% compared to 4.8% for those aged 18-24) [34]. This is alarming given that older adults are at the greatest risk of morbidity and mortality from smoking-related disease.

Population-level reductions in smoking prevalence may obscure age-related inequities, particularly when the primary measure of success is declining youth uptake. Without strategies that meaningfully support transition away from cigarettes among established adults who smoke, tobacco control risks overlooking the populations bearing the highest disease burden.

Health Status

Evidence also indicates that smoking is disproportionately concentrated among people living with disabilities. A large multi-country analysis using UNICEF-supported Multiple Indicator Cluster Surveys examined associations between disability and tobacco use among adults aged 18-49 years across 31 low- and middle-income countries [35]. Across pooled analyses, adults with disabilities were significantly more likely to use smoked tobacco products than those without disabilities, even after adjusting for age, education, household wealth, marital status, and residence. The magnitude of this association varied by country and sex, with particularly strong disparities observed among women with disabilities.

The same pattern is observed in high-income countries. The 2022 NHIS data revealed clear differences among Americans with disabilities: 18.6% smoked compared to 10.9% among those without a disability [34]. A nationally representative survey of more than a million adults in the UK (2017-2023) identified disparities in smoking status between disabled and non-disabled groups (current smoking prevalences of 17.5% and 11.7%, respectively) that persisted after adjusting for sociodemographic characteristics [36].

Current tobacco control strategies fail to account for these realities. Approaches built around taxation and restrictions may have reduced smoking uptake in higher-income, educated groups, but they have had diminishing returns among individuals with fewer resources. For people already facing economic and educational marginalization, increased taxes can worsen financial strain without providing meaningful pathways to cessation. Likewise, abstinence-only messaging and restrictive regulatory environments do little to support people whose smoking is intertwined with chronic stress, low health literacy, or limited access to alternative nicotine products. These inequities highlight an urgent need for more nuanced, person-centered strategies that recognize that the people who continue to smoke are not a homogeneous group but rather those for whom current policy has delivered the least benefit.

Implications for medical education and clinical practice

MPOWER’s abstinence-only orientation and the persistent conflation of nicotine with tobacco-related harm have limited its effectiveness. The framing of nicotine as inherently harmful, coupled with policies that restrict access to lower-risk nicotine products, has reinforced misinformation [37]. This has led to a harm-reduction gap that leaves many people who smoke without adequate support or access to accurate information and practical tools to transition away from the most harmful form of nicotine delivery.

The impact of this misframing is evident in both medical education and clinical practice. While smoking is rightly framed as the number one preventable cause of death and disease, and future physicians do learn about the epidemiology of tobacco-related harms, they remain strikingly ill-equipped to discuss smoking cessation or harm-reduction strategies. The result is that when people who smoke ask about non-combustible alternatives, many clinicians lack the competence or conviction to offer informed guidance. Curricula often emphasize what smoking does but insufficiently cover how to help people who smoke transition away from cigarettes when abstinence is unlikely.

For example, a National Health Service survey found that 61% of Scottish medical students reported that e-cigarettes were not mentioned at all in their curriculum, and 98% were unaware of any cessation programs [38]. A national mail survey of more than 1,000 U.S. physicians revealed that 83.2%, 80.9%, and 80.5% “strongly agreed” that nicotine directly contributes to the development of cardiovascular disease, chronic obstructive pulmonary disease, and cancer, respectively [39]. A cross-sectional study reported that 63% of Swiss physicians and pharmacists surveyed opposed recommending e-cigarettes as a cessation aid [40]. This is despite increasing patient interest and high-certainty evidence that these products increase quit rates [41]. A systematic review of general practitioners worldwide showed that most lacked the knowledge or confidence to counsel on or recommend e-cigarette alternatives for smoking cessation, despite being increasingly queried by patients [42].

There are some signs that this may be changing. A 2026 publication described consensus recommendations developed by 23 subject-matter experts and people in Canada with lived experience [43]. They are intended to guide healthcare practitioners, public health professionals, and members of the public in balancing individual harm reduction with population-level risk. Many more examples of such guidance are available on the Safer Nicotine Wiki [28].

Persisting with an abstinence-only paradigm risks perpetuating the very harms tobacco control seeks to eliminate. Reframing nicotine products as behavior-change agents or tools for transition (instead of the latest enemy) would enable clinicians, educators, and policymakers to adopt strategies that meet people who smoke where they are, rather than where we wish them to be.

Further progress in reducing smoking rates will require embracing innovation, informed choice, and a more nuanced understanding of nicotine dependence. It should empower people who smoke to make changes and prioritize health outcomes over ideology.

An EMPOWER-oriented approach should not be viewed as a simple substitute for existing tobacco control measures but as a complementary framework that warrants careful implementation and ongoing evaluation. Acknowledging potential limitations and unintended consequences is important for ensuring that efforts to reduce smoking-related harm remain balanced, equitable, and grounded in public health goals.
